# Increased expression of the TLR7/9 signaling pathways in chronic active EBV infection

**DOI:** 10.3389/fped.2022.1091571

**Published:** 2022-12-21

**Authors:** Luyao Liu, Ying Wang, Wenjie Wang, Wenjing Ying, Bijun Sun, Xiaochuan Wang, Jinqiao Sun

**Affiliations:** Department of Clinical Immunology, Children’s Hospital of Fudan University, National Chlidren’s Medical Center, Shanghai, China

**Keywords:** epstein-Barr virus, chronic active EBV infection, immune response, toll-like receptor, excessive inflammatory response

## Abstract

We aimed to investigate the immunological mechanisms of the Toll-like receptor (TLR) signaling pathways in different types of Epstein-Barr virus (EBV) infection. We retrospectively summarized the clinical data, routine laboratory tests and the immunological function of the infectious mononucleosis (IM) and chronic active EBV infection (CAEBV) patients. A real-time quantitative PCR array was used to detect the mRNA expression levels of TLR7/TLR9 and myeloid-differentiation factor 88 (MyD88). Flow cytometry was used to detect the protein expression of TLR7/TLR9. The MyD88 and nuclear factor-κB (NF-κB) (p65) protein were detected by western blotting. A cytometric bead array (CBA) assay was used to detect the expression of downstream cytokines. CAEBV patients presented with increased expression of TLR7/TLR9 in monocytes and B lymphocytes. TLR9 expression in the B lymphocytes of IM patients was decreased compared with the CAEBV pateints. Downstream signaling mediators, including MyD88 and NF-κB, were revealed to be increased in EBV-infected patients. Moreover, the expression of MyD88 and NF-κB was higher in CAEBV patients, leading to disrupted balance of downstream cytokines. EBV may activate the immune system *via* TLR7/TLR9 signaling pathways. Moreover, the overactivated TLR7/TLR9 pathway in CAEBV patients resulted in excessive inflammation, which might be relevant to the poor prognosis.

## Introduction

Epstein-Barr virus (EBV), a human gammaherpesvirus, infects 90%–95% of the world's human population. EBV infection has been found to be involved in the development of various human diseases from asymptomatic to malignant forms. Infectious mononucleosis (IM), one of the benign diseases associated with EBV infection, mainly causes transient fever, lymphadenopathy, and pharyngitis manifestations (1). Rare EBV-infected individuals without apparent immunodeficiency present with chronic active EBV infection (CAEBV), which is characterized by persistent or recurring IM-like symptoms, lymphadenopathy and in some patients hypersensitivity to mosquito bites and hydroa vacciniforme-like eruption of high EBV-DNA load in peripheral blood mononuclear cells (2). This disease can present with persistent or even fulminant expression with death and a poor prognosis (1). Most notably, EBV-associated diseases are relatively different in infected individuals, which might result from the presence of effective immune responses against EBV (3, 4).

Toll-like receptors (TLRs) can recognize EBV and initiate immune responses, which lead to the production of inflammatory cytokines and antiviral mediators. TLR7 and TLR9 are mainly expressed in intracellular vesicles. TLR7 is able to recognize EBV single-stranded RNA (ssRNA), whereas TLR9 recognizes EBV unmethylated 2′-deoxyribo (cytidine-phosphate-guanosine) (5). Myeloid differentiation factor 88 (MyD88) is recruited by TLRs and subsequently activates the transcription factor nuclear factor-κB (NF-κB) (6). NF-κB, as a transcription factor of REL family members, mediates inflammatory and antiapoptotic molecular signals. After activation, NF-κB can translocate to the nucleus and bind DNA to regulate downstream gene expression (2, 7, 8). Interactions between EBV and TLR signaling pathways might affect EBV infection and host antiviral immunity (5).

The molecular mechanism of TLR signaling pathways against EBV infection also remains to be fully revealed. In this study, we investigated the role of TLR signaling pathways in different EBV-associated diseases. Our results demonstrate that TLR7/9-MyD88-NF-κB pathways are overactivated in CAEBV patients, leading to excessive cytokine production.

## Methods

The study was approved by the Ethics Committee of the Children's Hospital of Fudan University. The patients and their parents provided written informed consent for enrollment in this study. The clinical trial registration number is NCT03374566 (12/12/2017-03/11/2021).

### Patients and clinical data

We included 10 healthy control children without EBV infection (ang-matched healthy control group), 20 children with acute infectious mononucleosis (IM group), and 10 children with CAEBV without a clear genetic defect (CAEBV group) in the study. The gender (M/F) of the groups was balanced. We summarized the clinical data and some related laboratory examinations of the patients.

### TLR7/9 signaling pathway detection by quantitative real-time PCR and flow cytometry

For real-time PCR, PBMCs were prepared from EDTA-treated whole blood from 10 healthy controls, 20 IM patients, and 10 CAEBV patients as previously reported (9). Total RNA was extracted from PBMCs using RNAiso Plus Reagent (TaKaRa, Japan), and cDNA was acquired using a reverse transcription kit (Qiagen, USA) following the manufacturer's instructions. Real-time RT-PCR was performed using Takara SYBR Fast qPCR Mix (Takara, Japan) on a LightCycler 480 Instrument II (Roche, Switzerland). The primer sequences were as follows: TLR7 (Forward): TGCTGTGTGGTTTGTCTGGT, TLR7 (Reverse): GCCCCACACAAGTCACATCT; TLR9 (Forward): CTGGCTGTTCCTGAAGTCTGTGC, TLR9 (Reverse): GTGGATGCGGTTGGAGGACAAG; MyD88 (Forward): AGTGGGATGGGGAGAACAGA, MyD88 (Reverse): TGTAGTCCAGCAACAGCCAG. Fold changes in the patients vs. healthy controls were analyzed using the 2 −ΔΔCT method. The β-actin gene was used as an inner reference.

For PhosFlow staining, we used a FACSCalibur flow cytometer (Becton Dickinson, Franklin Lakes, NJ, USA) for analysis (9). Whole blood was used for staining for lymphocyte surface markers after red cell lysis and analysis according to a standard multicolor flow cytometric protocol with appropriate fluorochrome-labeled monoclonal antibodies or isotype-matched control antibodies. After being washed twice with PBS, 1 × 104 to 5 × 104 live cells were analyzed by flow cytometry (Becton Dickinson, Franklin Lakes, NJ, USA) using FACSDiva software (BD Biosciences). Whole blood was permeabilized with Perm Buffer III according to the manufacturer's instructions (BD Biosciences). An APC-H7-conjugated antibody against CD3, a BV510-conjugated antibody against CD4, a PercP-Cy5-5-conjugated antibody against CD8, a BV421-conjugated antibody against CD14, and a PercP-Cy7-conjugated antibody against CD19 were used to gate lymphocyte subsets. For the Flow analysis, we used the following antibodies: PE-conjugated antibody against TLR7 and APC-conjugated antibody against TLR9 (all from BD Biosciences).

### Western blotting

As previously reported (10), cytoplasmic and nuclear proteins were extracted using NE-PERNuclear and Cytoplasmic Extraction Reagents (Thermo Fisher Scientific, USA) following the manufacturer's instructions. Equal amounts of cytoplasmic or nuclear extracts were separated on 12% SDS polyacrylamide gels and transferred to PVDF membranes. Blots were probed with primary antibodies against MyD88, NF-κB p65, β-actin, or histone H3 (Cell Signaling Technology, Beverly, MA). Primary antibodies were detected with horseradish peroxidase-conjugated secondary antibody. Visualization was performed using an ECL peroxidase substrate.

### Cytokine detection

We used a FACSCalibur flow cytometer (Becton Dickinson, Franklin Lakes, NJ, USA) to measure cytokine expression using a BD Cytometric Bead Array (CBA) Human Soluble Protein Master Buffer Kit (11). We first added 50 µl of flex set standard dilutions to the first 8 tubes (no standard dilution, 1:729, 1:243, 1:81, 1:27, 1:9, 1:3 and top standard). We then added 50 µl of each unknown sample to the appropriate assay tubes and added 20 µl of the mixed capture beads to each assay tube. The tubes were incubated for 2 h at room temperature. Twenty microliters of the mixed Human Detection Reagent (Part A) were added to each assay tube. The tubes were incubated for 2 h at room temperature. We added 1 ml of wash buffer to each assay tube with centrifugation at 200 g for 5 min. Then, we added 100 µl of enhanced sensitivity detection reagent (Part B) to each assay tube with gentle mixing of the tubes. The tubes were incubated for 1 h at room temperature. Then, 300 µl of wash buffer was added to each assay tube with brief vertexing to resuspend the beads. All reagents were from BD Biosciences. Finally, we used FCAP array software to analyze the data.

### Statistical analysis

The data were analyzed using Student's *t*-tests, Welch tests, or nonparametric Mann–Whitney *U* tests with GraphPad Prism software; *p*-values <0.05 were considered significant.

## Results

### Clinical characteristics

The clinical manifestations of children with EBV infection are shown in Supplementary Table S1. Fever is the most common clinical manifestation. Thirty children (100%) with EBV infection all presented with fever. Lymphadenopathy (95% IM vs. 80% CAEBV), hepatomegaly (80% IM vs. 60% CAEBV), and splenomegaly (80% IM vs. 60% CAEBV) were observed in the patients. Among the patients, ten IM patients (50%) with hepatomegaly presented with liver dysfunction, while the six CAEBV patients described above all (100%) had liver dysfunction. Bilateral eyelid edema was observed in 11 (55%) IM children, whereas none was observed in the CAEBV group. Two CAEBV children (P25 and P30) developed hemophagocytic syndrome presenting with continual fever and pancytopenia.

### Laboratory examinations

EBV VCA IgM in blood serum was positive in all IM patients, whereas only one CAEBV case was positive for EBV VCA IgM. On the other hand, seventeen children in the IM group (85%) had elevated EBV VCA IgG, and in the CAEBV group, there were nine patients (90%). Moreover, the increase in the EBV VCA IgG level in the CAEBV children (mean = 153.44 ± 65.2) was much greater than in the IM children (mean = 83.7 ± 52.4). We found that in thirteen IM patients (65%), the number of white blood cells (WBCs) was increased, whereas the levels of WBCs in six CAEBV children (60%) were decreased.

The immunological characteristics of the patients are listed in Supplementary Table S2. We observed that EBV-infected patients presented with elevated IgG and IgE levels (70% IM vs. 70% CAEBV, 30% IM vs. 30% CAEBV, respectively). However, the CAEBV patients had significantly higher IgE levels, of more than 1,000 KU/L. Increased CD8+ T lymphocytes (95%) and decreased CD4+ T lymphocytes (70%)/B lymphocytes (90%)/NK cells (80%) were observed in the IM patients. Compared with the children in the IM group, the counts of T lymphocytes were significantly lower in the CAEBV group. The proportion of CD8+ T cells in four CAEBV children (40%) was increased, while it was in the normal range in 5 patients (50%). Importantly, nine patients (90%) had a reduced proportion of B lymphocytes. With regard to NK cells, a reduced proportion was observed in four patients (40%), while an increased proportion was observed in three patients (30%).

### Expression of the TLR7/TLR9 - MyD88-Nf-κB/IRF7 signaling pathway

#### TLR7 and TLR9 expression levels were increased in CAEBV infection

**Figure 1:**
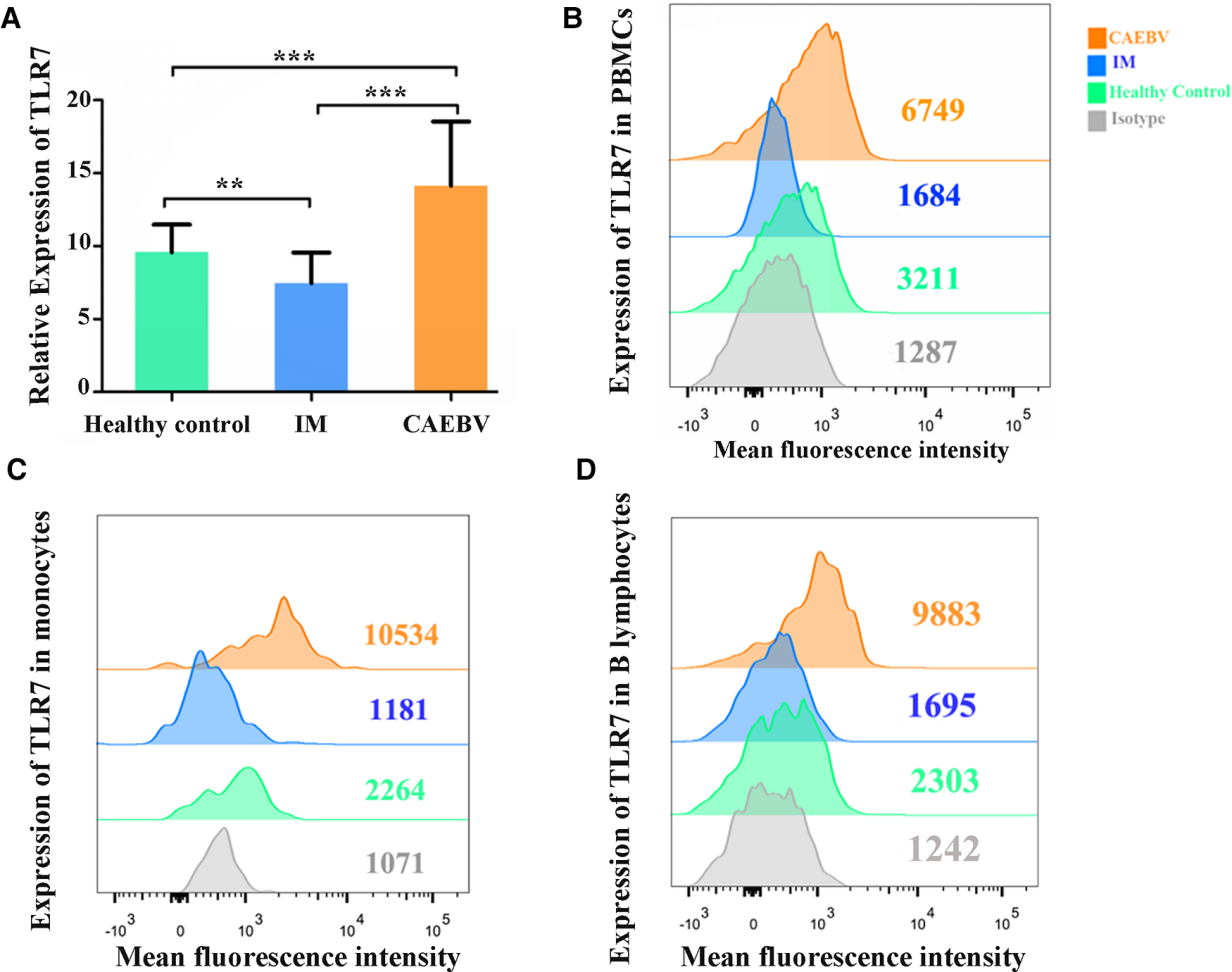
TLR7 expression is associated with EBV infection. (**A**) MRNA accumulation of TLR7 was measured in PBMCs from healthy controls (age matched, *n* = 20), IM patients (*n* = 20) and CAEBV patients (*n* = 10). (**B**) Flow cytometry analysis showed the levels of TLR7 protein. (**C**) The expression of TLR7 in CD19+ B lymphocytes by flow cytometry analysis. (**D**) The expression of TLR7 in CD14+ monocytes by flow cytometry analysis. (**p < 0.01 and ***p < 0.001 by Duncan's test).

**Figure 2:**
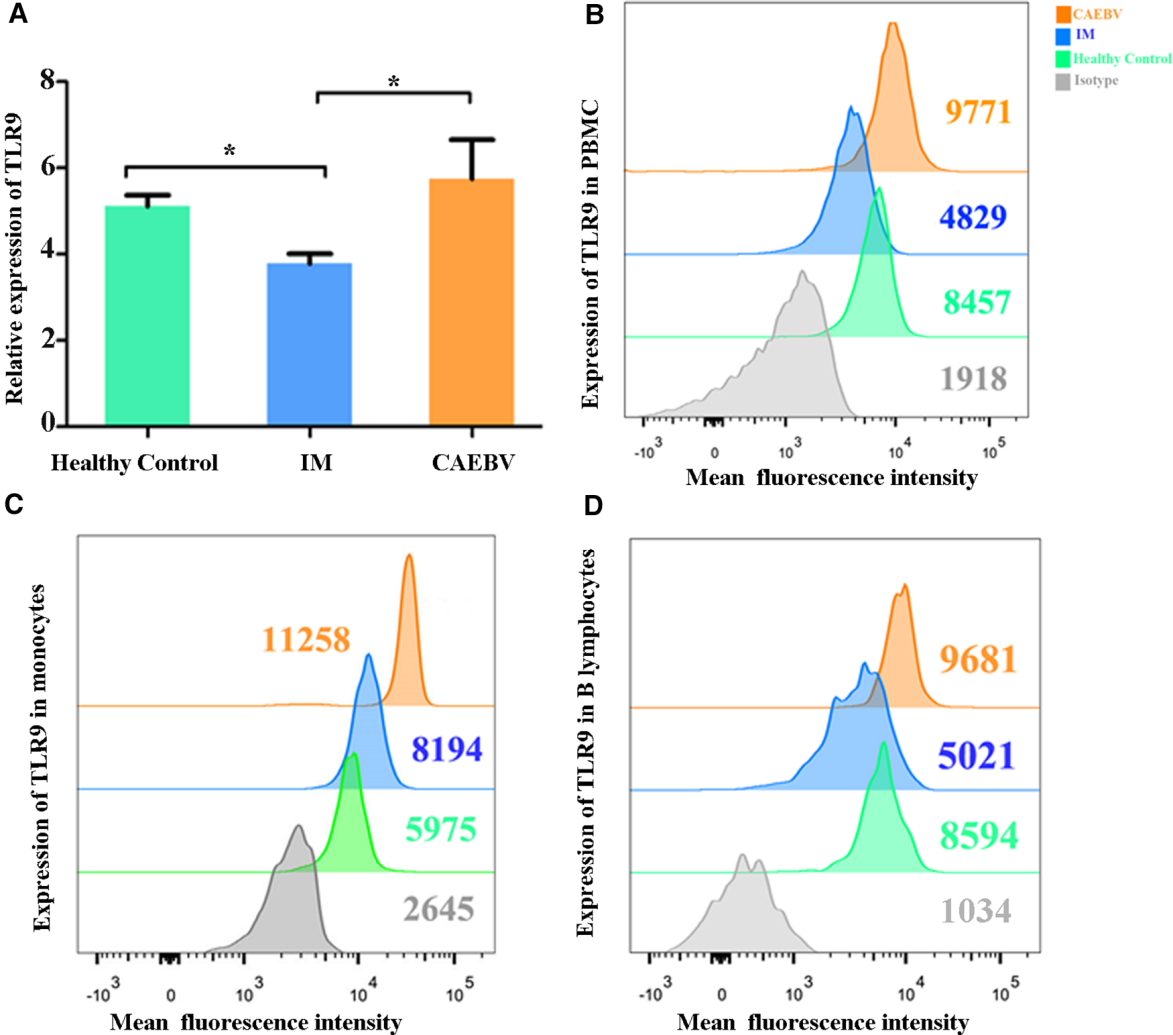
TLR9 expression is associated with EBV infection. (**A**) The level of TLR9 mRNA was measured in PBMCs from healthy controls (age matched, *n* = 20), IM patients (*n* = 20) and CAEBV patients (*n* = 10). (**B**) Flow cytometry analysis showed the levels of TLR9 protein. (**C**) The expression of TLR9 in CD19+ B lymphocytes by flow cytometry analysis. (**D**) The expression of TLR9 in CD14+ monocytes by flow cytometry analysis. (*p < 0.05 by Duncan's test).

Real-time PCR and flow cytometry analyses were performed to determine whether the expression of TLR7 and TLR9 was changed. We found that TLR7 and TLR9 transcriptional levels were significantly increased in peripheral blood mononuclear cells (PBMCs) of CAEBV patients compared with IM patients and healthy controls (Figures 1A, 2A). We therefore checked whether TLR7 and TLR9 protein levels were also altered in EBV-infected individuals. Moreover, the TLR7 and TLR9 protein levels were in accordance with the transcriptional levels (Figures 1B, 2B). The results suggested that TLR7 and TLR9 were activated in CAEBV patients. However, TLR7 and TLR9 expression in IM patients was downregulated in PBMCs. Flow cytometry analysis showed that the TLR7 and TLR9 protein levels were in accordance with the mRNA changes (Supplementary Figure S1A,D).

Moreover, we separately stained the surface markers CD19 and CD14 to gate B lymphocytes and monocytes and then investigated the expression of TLR7 and TLR9. We found that CAEBV patients presented with increased expression of TLR7 in CD14+ monocytes and CD19+ B lymphocytes (Figure 2C,D), whereas its expression in IM patients decreased compared to the CAEBV patients (Figure 1C). TLR9 expression in B lymphocytes and monocytes was significantly higher in CAEBV patients than in IM patients (Figure 1D). Our data demonstrated that the expression of TLR9 was downregulated in B lymphocytes of children with IM, whereas it was overexpressed in CD14+ monocytes (Supplementary Figure S1). These observations suggest that EBV influenced TLR9 expression, possibly depending on the cell type.

#### Activation of the TLR7/9 downstream signaling pathway

**Figure 3:**
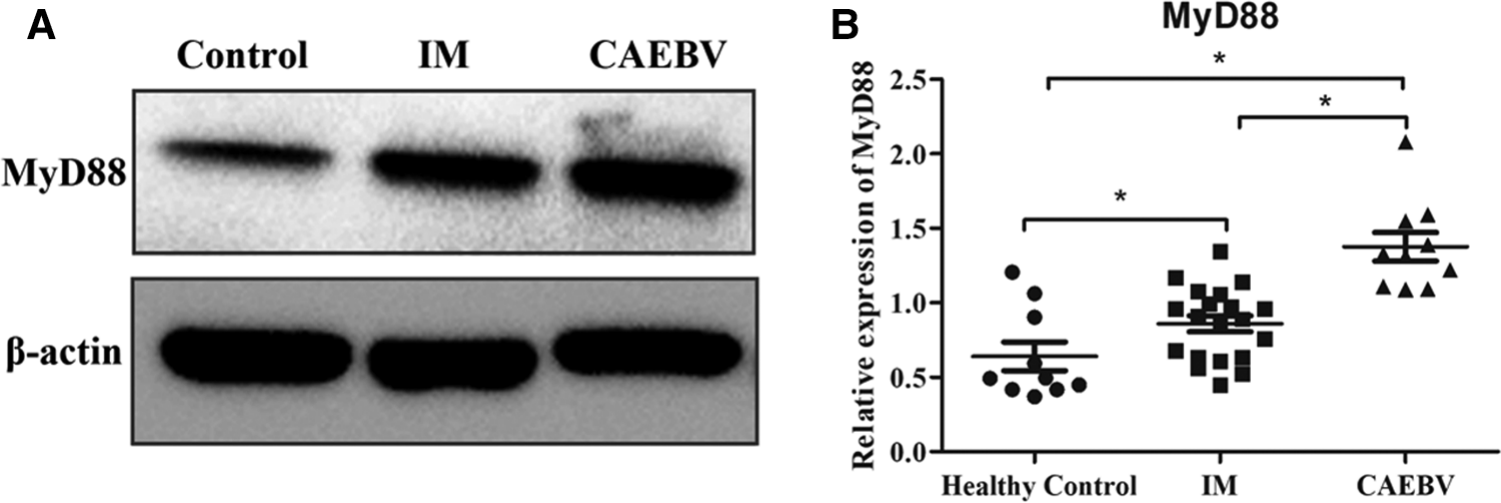
Myd88 expression in the EBV-infected patients. (**A**) The protein level of MyD88 in the cytosolic fractions derived from the healthy control, IM patient and CAEBV patient. (**B**) The protein level of MyD88 in healthy controls (age matched, *n* = 20), IM patients (*n* = 20) and CAEBV patients (*n* = 10). (*p < 0.05 by Duncan's test).

Signaling of TLR7 and TLR9 proceeds through MyD88, leading to the activation of the NF-κB signaling pathway (6). Western blot analysis showed that the expression levels of MyD88 were increased in EBV-infected patients compared with healthy controls. Furthermore, MyD88 expression in CAEBV patients was higher than that in IM patients, suggesting stronger activation of MyD88 in CAEBV than in IM patients (Figure 3A,B).

**Figure 4:**
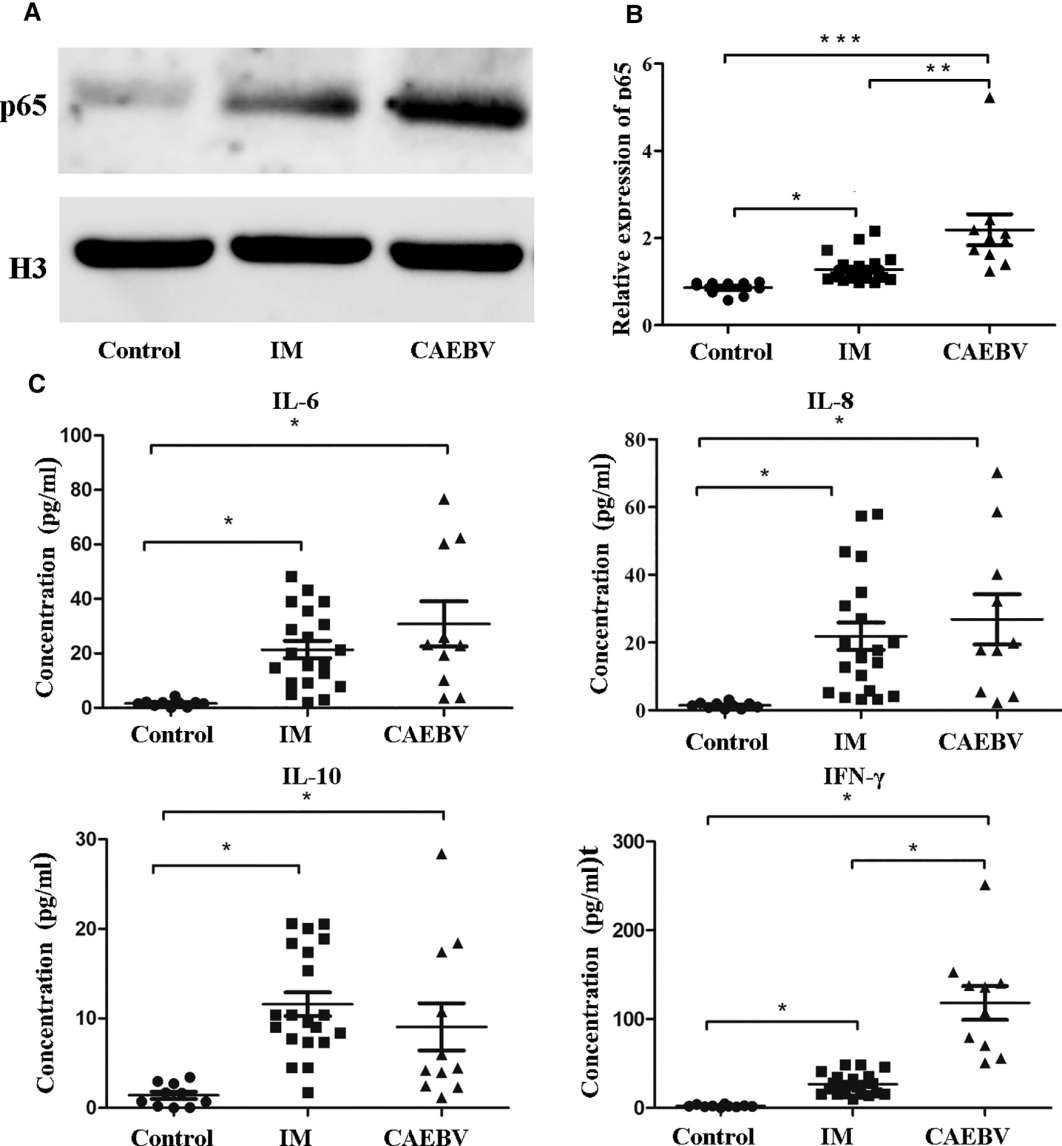
NF-κB signaling activation in EBV-infected patients. (**A**) The protein levels of p65 in the nucleus by Western blot analysis. (**B**) The protein level of p65 in the nucleus in healthy controls (age-matched *N* = 10), IM patients (*n* = 20) and CAEBV patients (*n* = 10). (**C**) Cytometric bead array (CBA) by flow cytometry showed that the expression levels of downstream cytokines, such as IL-6/IL-8/IL-10 and IFN-γ, were measured in serum obtained from healthy controls (age matched, *n* = 20) and IM patients (*n* = 20) and CAEBV patients (*n* = 10). (*p < 0.05 **p < 0.01 and ***p < 0.001 by Duncan's test).

Once activated, the free NF-κB molecular subunits, for example, p50 and p65, translocate into the nucleus to regulate the expression of multiple target genes involved in cell activation and the production of inflammatory cytokines (12, 13). To demonstrate the activation of the NF-κB signaling pathway, we monitored the expression of NF-κB p65 in the cell nucleus. As shown in Figure 4A, we detected that the p65 level in nuclei was significantly higher in EBV patients than in healthy controls. Furthermore, the CAEBV patients presented with prominently upregulated NF-κB p65 compared with the IM patients. These results indicated that the NF-κB signaling pathway was activated in EBV-infected patients, particularly in CAEBV patients.

#### Increased expression of cytokines after EBV infection

Once TLRs are activated, immunocytes rapidly produce a broad range of inflammatory cytokine responses against EBV infection (5, 14). Consistent with the aforementioned laboratory results, we detected cytokines such as IL-6, IL-8, IL-10 and IFN-γ in the serum. The expression levels of cytokines in EBV-infected patients were observed to increase compared with those in healthy controls (Figure 4C). In addition, the expression levels IFN-γ in the CAEBV group were observed to be significantly greater than those in the IM group.

## Discussion

Epstein-Barr virus (EBV), a human herpesvirus, maintains a finely balanced relationship with the host; thereafter, most carriers are asymptomatic during their lifetime. Some individuals occasionally develop IM, one of the benign and acute diseases, after infection with EBV. Moreover, a minority of EBV-infected individuals present with CAEBV characterized by a poor prognosis (1). Large expansions of CD8+ T lymphocytes are detected to control EBV infection during IM (15). We observed that CD8+ T lymphocytes were significantly expanded in children with IM. In addition, in CAEBV patients, EBV-specific CD8+ T lymphocytes and NK cells were detected to be lower than those in IM patients, making it difficult to control EBV infection. CD8+ T lymphocyte and NK cell deficiency might be associated with loss of control of EBV infection. The CAEBV children showed higher expression of EBV-VCA IgG than the IM children, which might result from continuously uncontrolled EBV infection.

The interaction between EBV infection and the immune system by TLRs plays a critical role in the outcome of the infection. The present study (5) demonstrated that EBV can develop complex strategies to interact with TLR signaling pathways. NF-κB, the transcription factor related to innate immune response genes, has recently been found to be overexpressed in cells infected with EBV (2, 16). Appropriate production of cytokines might be beneficial for EBV infection; however, excessive cytokines might have a negative impact on EBV-infected hosts. Dysregulation of TLR-mediated immune responses might be related to autoimmunity or autoinflammatory diseases by inducing abnormal activation of host immunity (17). The expression of TLR7 and TLR9 signaling pathways can be elicited or modulated by EBV infection (17). The role of the TLR signaling pathway in the fate of EBV infection has remained undermined. Farina et al. (18) found that expression of the EBV lytic gene BFRF1 was associated with a trend of TLR7 downregulation. In vitro studies showed that EBV can upregulate the expression of TLR7 and MyD88 in B lymphocytes (19). In our study, we evaluated the role of TLR7 and TLR9 in different EBV infections. Our results demonstrated that TLR7 expression levels in CAEBV patients were significantly increased, including CD14+ monocytes and CD19+ B lymphocytes, whereas TLR7 expression in IM patients was decreased. Therefore, we presume that the different stages of EBV-related proteins might affect the expression level of TLR7. In contrast, TLR7 expression in CAEBV patients remained high, which might be relevant to individual CAEBV specificity. The same phenomenon occurs in the expression of TLR9. Compared with children in the IM group, the expression of TLR9 in children with CAEBV was increased, both in monocytes and B lymphocytes. However, our data demonstrated that the expression of TLR9 was downregulated in B lymphocytes in children with IM, whereas it was overexpressed in CD14+ monocytes. These results indicated that EBV infection leads to an increase in TLR9 expression in monocytes. The impact of EBV infection with different TLR expression might depend on cell and individual specificity. Van Gent et al. identified that the EBV lytic-phase protein BGLF5 can downregulate TLR9 levels *via* RNA degradation *in vitro* (20). In addition, Fathallah et al. found that EBV latent membrane protein 1 (LMP1) can prevent TLR9 promoter deregulation and inhibit TLR9 transcription by activating the NF-κB pathway in B lymphocytes (21). Our data demonstrated that MyD88 and NF-κB are overexpressed in EBV-infected individuals compared to controls. TLR7 and TLR9 signaling themselves mediate NF-κB activation, inducing a negative feedback loop for TLR9 expression in B lymphocytes. Thus, EBV-induced NF-κB activity might be the source of TLR9 downregulation in B lymphocytes, which might be related to cell specificity. Moreover, TLR9 can physically interact with TLR7 and inhibit TLR7 function in a dose-dependent manner (22). However, CAEBV patients developed persistently excessive activation of TLR7 and TLR9 and its downstream signaling mediators. Our present results indicate that CAEBV patients might develop deficiency in the self-regulation of TLR negative feedback.

NF-kB is constitutively activated in many types of viral and nonviral-associated human cancers. Activation of the NF-κB signaling pathway induces NF-kB nuclear translocation and affects the expression of genes encoding key players in cell survival, cellular proliferation, and the immune response, which result in the production of downstream cytokines. As a result, its downstream signaling cytokines were upregulated. The upstream molecules TLR7 and TLR9, whose expression is upregulated in CAEBV patients, might play indirect roles in NF-κB activation. Previous studies have found that EBV upregulates the expression of IL-6, IL-8 and IFN-γ by activating the TLR9 signaling pathway (4, 23). IL-10, as an immunosuppressive factor, is mainly secreted by monocytes and Th1-type cells. It can regulate cell growth and differentiation and participate in a variety of inflammatory and immune responses. TLR9 has been found to promote the differentiation of T cells into Th1 cells and participate in the synthesis and secretion of IL-10. Moreover, TLR9 is also involved in the process of secretion of IFN-γ by T lymphocytes (24). Excessive activation of NF-κB signaling has been linked to human inflammatory diseases (25). We observed that high levels of human cytokines, including IL-6, IL-8, IL-10, and IFN-γ, were detected in the blood serum of EBV-infected patients. Moreover, the expression of IL-10 and IFN-γ increased significantly in CAEBV patients. The TLR signaling pathway, which is related to the secretion of multiple cytokines, plays complicated roles in EBV infection. The changes in the adaptor MyD88 and mediator NF-κB were inconsistent with TLR expression in IM patients. We suspected that, on the one hand, in the early stage of EBV infection, TLR expression was upregulated, which then downregulated negative feedback. On the other hand, EBV leads to increased expression of MyD88 and NF-κB *via* other signaling pathways. Our results demonstrated that CAEBV patients maintain excessive inflammatory conditions, mirroring hypercytokinemia characteristics of CAEBV patients. TLR7 and TLR9 signaling pathways may have distinct outcomes depending on the cell types expressing them. They are critically involved not only in the activation of immunocytes but also in cytokine induction.

Both chronic infection and excessive inflammation contribute to the occurrence and development of tumors (26). Nakamura et al. reported that the expression of cytosine deaminase (AID), which participates in somatic hypermutation and the transformation of immunoglobulin classes, was upregulated in CAEBV patients. The dysregulation of AID leads to gene mutations and B-cell lymphoma (2). NF-κB signaling has been found to induce the expression of AID (27). Therefore, overactivation of the TLR-NF-κB pathway may play a certain role in the development of EBV-related lymphoma. Dysregulated AID expression induces genomic mutation, leading to the development of B cell lymphoma. Interestingly, NF-κB can induce AID expression in B cells. The activation of NF-κB in CAEBV patients may lead to the upregulation of AID, which might be prone to develop lymphomagenesis.

## Conclusion

Our study provides new insights into the possible involvement of TLR7 and TLR9 in the pathogenesis of CAEBV, and TLR7/9-dependent immune responses to EBV might contribute to the long-term inflammatory response in CAEBV patients. Here, we provide evidence that excessive inflammatory responses meditated by overexpression of the TLR7/TLR9-MyD88-NF-κB signaling pathway might contribute to the poor prognosis of CAEBV. Nevertheless, additional mechanistic investigations are need to definite how the TLR7/TLR9-MyD88-NF-κB signaling pathway becomes over activated.

## Data Availability

The raw data supporting the conclusions of this article will be made available by the authors, without undue reservation.

## References

[1] Okano M. Recent concise viewpoints of chronic active epstein-barr virus infection. Curr Pediatr Rev. (2015) 11(1):5–9. 10.2174/1573396311666150501002809 25938379

[2] Takada H Imadome K-I Shibayama H Yoshimori M Wang L Saitoh Y EBV Induces persistent NF-*κ*B activation and contributes to survival of EBV-positive neoplastic T- or NK-cells. PLoS ONE. (2017) 12(3):e174136. 10.1371/journal.pone.0174136 PMC5367708 28346502

[3] Hislop AD TG. T-Cell responses to EBV. Curr Top Microbiol Immunol. (2015) 391:325–53. 10.1007/978-3-3319-22834-1_11 26428380

[4] Lunemann A Rowe M Nadal D. Innate immune recognition of EBV. Curr Top Microbiol Immunol. (2015) 391:265–87. 10.1007/978-3-319-22834-1_9 26428378

[5] Zauner L Nadal D. Understanding TLR9 action in Epstein-Barr virus infection. Front Biosci. (2012) 17(4):1219–31. 10.2741/3982 22201799

[6] Kawai T Akira S. The role of pattern-recognition receptors in innate immunity: update on Toll-like receptors. Nat Immunol. (2010) 11(5):373–84. 10.1038/ni.1863 20404851

[7] Baldwin AS. Series Introduction: the transcription factor NF-κB and human disease. J Clin Invest. (2001) 107(1):3–6. 10.1172/JCI11891 11134170 PMC198555

[8] Karin M Cao Y Greten FR Li Z. NF-κB in cancer: from innocent bystander to major culprit. Nat Rev Cancer. (2002) 2(4):301–10. 10.1038/nrc780 12001991

[9] Wang Y Wang W Liu L Hou J Ying W Hui X Report of a Chinese cohort with activated phosphoinositide 3-kinase δ syndrome. J Clin Immunol. (2018) 38(8):854–63. 10.1007/s10875-018-0568-x 30499059

[10] Dong X Liu L Wang Y Yang X Wang W Lin L Novel heterogeneous mutation of TNFAIP3 in a Chinese patient with behçet-like phenotype and persistent EBV viremia. J Clin Immunol. (2019) 39(2):188–94. 10.1007/s10875-019-00604-9 30810840

[11] Liu L Wang W Wang Y Hou J Ying W Hui X A Chinese DADA2 patient: report of two novel mutations and successful HSCT. Immunogenetics (New York). (2019) 71(4):299–305. 10.1007/s00251-018-01101-w 30610243

[12] Hayden MS Ghosh S. Signaling to NF-kappaB. Genes Dev. (2004) 18(18):2195–224. 10.1101/gad.1228704 15371334

[13] Takeshita F Gursel I Ishii KJ Suzuki K Gursel M Klinman DM. Signal transduction pathways mediated by the interaction of CpG DNA with Toll-like receptor 9. Semin Immunol. (2004) 16(1):17–22. 10.1016/j.smim.2003.10.009 14751759

[14] Fiola S Gosselin D Takada K Gosselin J. TLR9 Contributes to the recognition of EBV by primary monocytes and plasmacytoid dendritic cells. J Immunol. (2010) 185(6):3620–31. 10.4049/jimmunol.0903736 20713890

[15] Taylor GS Long HM Brooks JM Rickinson AB Hislop AD. The immunology of epstein-barr virus-induced disease. Annu Rev Immunol. (2015) 33:787–821. 10.1146/annurev-immunol-032414-112326 25706097

[16] Younesi V Shirazi FG Memarian A Amanzadeh A Jeddi-Tehrani M Shokri F. Assessment of the effect of TLR7/8, TLR9 agonists and CD40 ligand on the transformation efficiency of Epstein-Barr virus in human B lymphocytes by limiting dilution assay. Cytotechnology. (2014) 66(1):95–105. 10.1007/s10616-013-9542-x 23404520 PMC3886530

[17] Cavalcante P Galbardi B Franzi S Marcuzzo S Barzago C Bonanno S Increased expression of Toll-like receptors 7 and 9 in myasthenia gravis thymus characterized by active Epstein–Barr virus infection. Immunobiology. (2016) 221(4):516–27. 10.1016/j.imbio.2015.12.007 26723518

[18] Farina A Peruzzi G Lacconi V Lenna S Quarta S Rosato E Epstein-Barr virus lytic infection promotes activation of Toll-like receptor 8 innate immune response in systemic sclerosis monocytes. Arthritis Res Ther. (2017) 19(1):39. 10.1186/s13075-017-1237-9 28245863 PMC5331713

[19] Martin HJ Lee JM Walls D Hayward SD. Manipulation of the toll-like receptor 7 signaling pathway by epstein-barr virus. J Virol. (2007) 81(18):9748–58. 10.1128/JVI.01122-07 17609264 PMC2045431

[20] van Gent M Griffin BD Berkhoff EG van Leeuwen D Boer IGJ Buisson M EBV lytic-phase protein BGLF5 contributes to TLR9 downregulation during productive infection. J Immunol. (2011) 186(3):1694–702. 10.4049/jimmunol.0903120 21191071

[21] Fathallah I Parroche P Gruffat H Zannetti C Johansson H Yue J EBV Latent membrane protein 1 is a negative regulator of TLR9. J Immunol. (2010) 18(11):6439–47. 10.4049/jimmunol.0903459 20980631

[22] Wang J Shao Y Bennett TA Shankar RA Wightman PD Reddy LG. The functional effects of physical interactions among Toll-like receptors 7, 8, and 9. J Biol Chem. (2006) 281(49):37427–34. 10.1074/jbc.M605311200 17040905

[23] Imadome K-i Yajima M Arai A Nakazawa A Kawano F Ichikawa S Novel mouse xenograft models reveal a critical role of CD4+ T cells in the proliferation of EBV-infected T and NK cells. PLoS Pathog. (2011) 7(10):e1002326. 10.1371/journal.ppat.1002326 22028658 PMC3197618

[24] Lim WH Kireta S Russ GR Coates PT. Human plasmacytoid dendritic cells regulate immune responses to Epstein-Barr virus (EBV) infection and delay EBV-related mortality in humanized NOD-SCID mice. Blood. (2007) 109(3):1043–50. 10.1182/blood-2005-12-024802 17018863

[25] Rui L Schmitz R Ceribelli M Staudt LM. Malignant pirates of the immune system. Nat Immunol. (2011) 12(10):933–40. 10.1038/ni.2094 21934679

[26] Balkwill F Coussens LM. Cancer: an inflammatory link. Nature. (2004) 431(7007):405–6. 10.1038/431405a 15385993

[27] Toussirot E Roudier J. Pathophysiological links between rheumatoid arthritis and the Epstein-Barr virus: an update. Joint Bone Spine. (2007) 5(74):418–26. 10.1016/j.jbspin.2007.05.001 17625943

