# Supplementary figures and images for: Increased expression of the TLR7/9 signaling pathways in chronic active EBV infection
Source: Front Pediatr. 2022 Dec 21;10:1091571. doi: 10.3389/fped.2022.1091571 (PMC9811674; doi:10.3389/fped.2022.1091571)

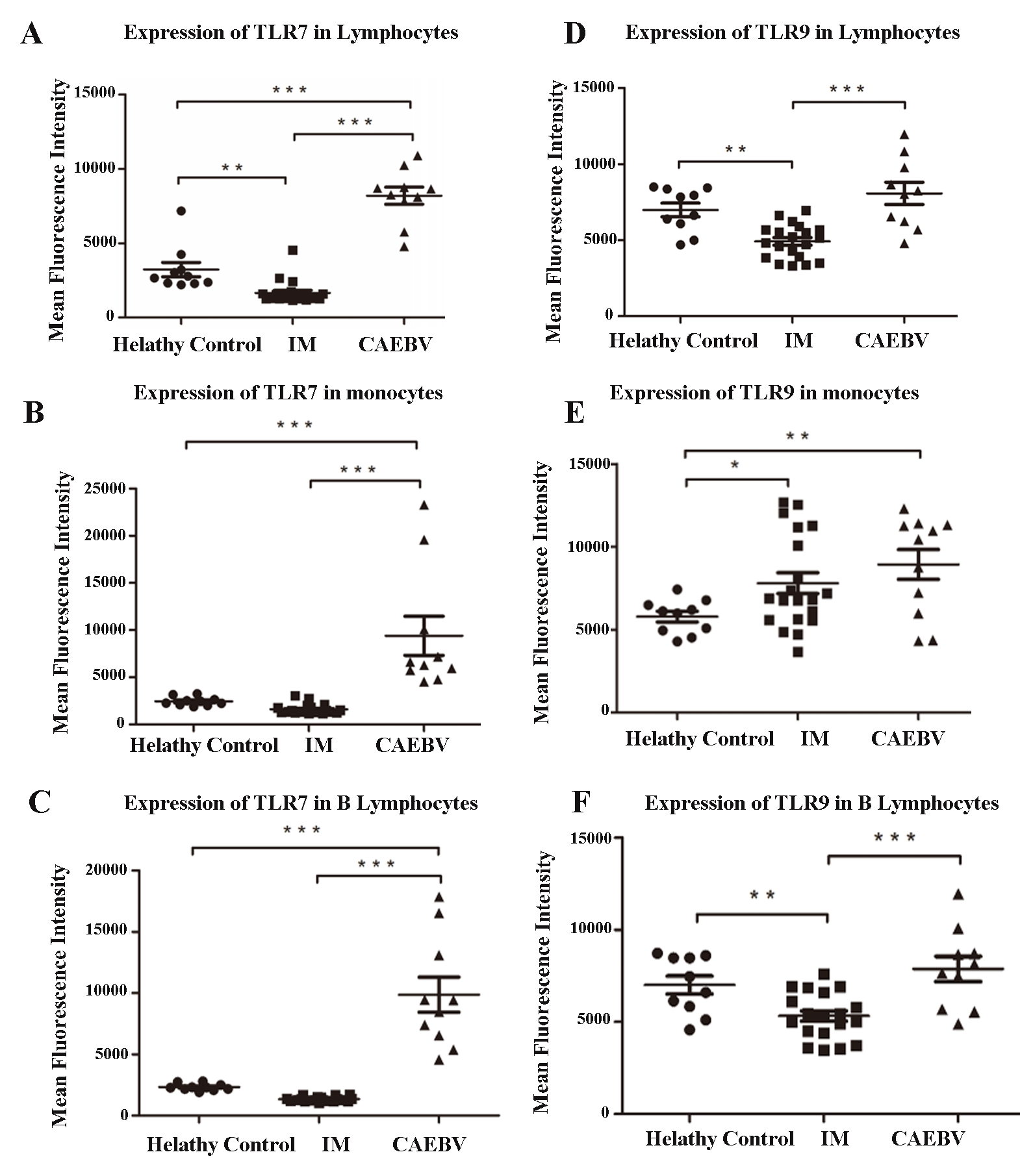

Supplement: Supplementary file 3 [file Image1.tif]
